# Idiopathic necrotizing fasciitis following fracture fixation: A case report

**DOI:** 10.1097/MD.0000000000020874

**Published:** 2020-06-26

**Authors:** Muhammad Umar Joomun, Zhiya Li, Deting Xue, Huawei Shao, Zhijun Pan

**Affiliations:** a Department of Orthopaedics, The Second Affiliated Hospital of Zhejiang University, School of Medicine, Hangzhou, Zhejiang Province; b Department of Emergency, The Second Affiliated Hospital, Guangzhou University of Chinese Medicine, Guangzhou, Guangdong Province; c Department of Burns and Wound Center, The Second Affiliated Hospital of Zhejiang University, School of Medicine, No. 88 Jiefang road, Hangzhou, Zhejiang Province, People's Republic of China.

**Keywords:** diabetes mellitus, idiopathic, intertrochanteric fracture, necrotizing fasciitis, wound management

## Abstract

**Introduction:**

Idiopathic necrotizing fasciitis (NF) is an infrequent, highly lethal skin infection that spreads rapidly, marked by fascia and subcutaneous tissue necrosis. It occurs in the absence of a known causative factor. Its emergence after sterile orthopedic fixation with unexpected spread to the abdomen may turn to be challenging both as a medical and surgical emergency.

**Patient concerns:**

A 56-year-old diabetic female presented with multiple fractures. After open reduction and internal fixation (ORIF) with iliac crest grafting of hip fracture, she developed incisional NF which later spread to the abdomen.

**Diagnosis:**

Post-ORIF of hip fracture complicated with idiopathic NF and abdominal spread.

**Interventions:**

She underwent emergency débridements with negative pressure wound therapy and broad-spectrum intravenous antibiotic therapy. After granulation, the wounds were closed with skin flaps and grafts with antibiotic beads. When the NF spread to the abdomen, additional débridements during abdominal explorations were performed.

**Outcomes:**

The patient was initially stable with promising healings of the wounds. Later, the patient suddenly developed a high fever and severe abdominal pain. Ultrasound revealed that NF emerged unexpectedly in the right lower abdomen. The causative agent of the NF remained undetected. Despite all the extensive treatments, the patient's condition deteriorated rapidly. She died of septic shock and multiple organ failure.

**Conclusion:**

The idiopathic NF may still potentially occur after a clean ORIF of the hip region. The implementation of intensive guideline-based treatments may show improvements, but the risk of unexpected NF spread to the abdomen should be anticipated, which may increase the mortality rates in diabetic or immunocompromised patients.

## 1 Introduction

Idiopathic necrotizing fasciitis (NF) of the extremities is a relatively rare but devastating infection. It is characterized by widespread necrosis which progresses rapidly along fascial planes and extends well beyond the perceivable signs of infection, which may occur without a known causative agent.^[1]^ The majority of the patients who develop NF have a history of minor or major trauma, insulin injections, surgical complications, and abscesses.^[2]^ Early recognition is vital because the disease can progress to massive tissue destruction, amputations, systemic toxicity, and even death. The laboratory risk indicator for NF scores (LRINEC score) based on the 6 serum parameters and the skin zoning classifications described by Wong CH et al^[3,4]^ remain as the vital tools in detecting and grading NF. Despite the improving diagnosis of NF and early surgical intervention, the reported mortality rates remain high (6% to 76%).^[5]^ The recognized risk factors include diabetes, advanced age, chronic immunosuppression, obesity, and underlying malignancy.^[6]^ Moreover, recent researches show that the most common comorbidity involved in the development of NF is diabetes mellitus (DM), presented in 44.5% to 72.3% of the cases.^[7]^ This paper presented and discussed a multidisciplinary case of idiopathic NF in a diabetic polytrauma patient after a sterile orthopedic surgery of the hip which unexpectedly spread to the abdomen. Up to date, there is no literature that has ever documented the idiopathic NF in similar settings.

## 2 Case presentation

During hospitalization, the patient has agreed and given her consent for her clinical and imaging details to be used for scientific publication. Unfortunately, before we requested her to sign the ‘Patient consent form’ required for the publication, the patient has passed away already and her relatives were out of reach. All efforts have been made to ensure that this case report has been completely anonymized, the images are unidentifiable and there are no personal details on the reported patient. The ethical approval form (Registration number: 2019–137) was obtained from the Ethical Committee of our hospital (Institutional Review Board of The Second Affiliated Hospital of Zhejiang University School of Medicine) on 12 May 2019.

A 56-year old female farmer, after a traffic collision, she complained of severe pain and swelling on the left lower extremity. She has been diabetic for 10 years and denied smoking, intravenous (IV) drug or alcohol abuse with no other significant past medical history. At the local hospital, the patient's primary diagnosis was multiple fracture injuries. She received left femoral supracondylar traction along with symptomatic treatments but subsequently developed a recurrent fever and skin ulceration of left leg and knee. Then, she was transferred to our Burn Surgery department for around 6 weeks. Here, the 5 interval surgeries were performed, consisting of débridements with negative pressure wound therapy (NPWT), neurovascular exploration, muscle flap transfer, and skin grafting. Post-operatively, the patient remained stable, received antibiotics, fluid replacement, daily dressings change. All the wound flaps and skin grafts survived.

**Figure 1:**
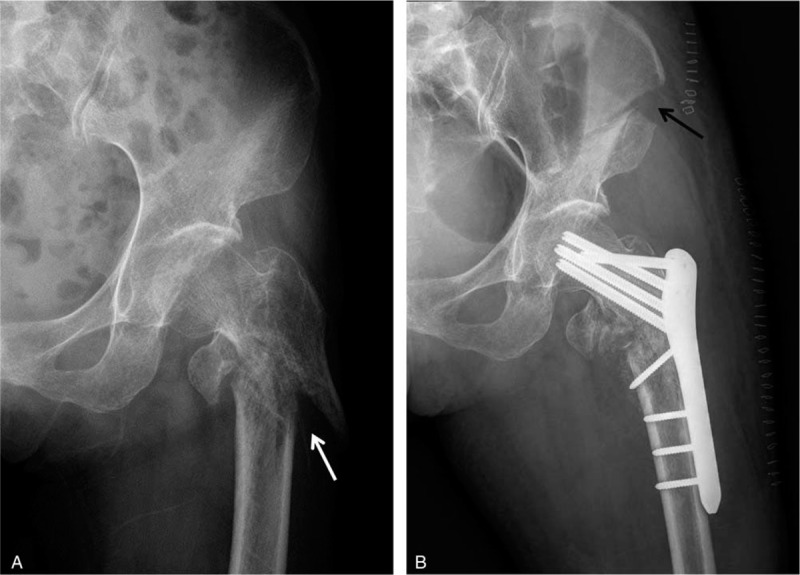
Plain radiography. (A) Delayed AO/OTA 31-A3 left femoral intertrochanteric fracture (white arrow). (B) Proximal femoral locking plate supplemented with anterior iliac crest bone graft harvested (black arrow).

One month later, since her general condition was excellent and the wounds healed well, the patient was transferred to our Orthopedics department (considered as ‘day 0’ for reference) for the AO/OTA 31-A3 left femoral intertrochanteric fracture. Physical examinations showed that the skin graft site of the left leg has already healed with no local redness or ulceration. There was a limited left hip and knee movement, but normal muscle tension and sensation of the left lower limb. The blood supply of the distal limb is adequate. Pathological signs were unremarkable. On day 4, open reduction and internal fixation (ORIF) was performed, using proximal femoral locking plate (PERI-LOC PFP, Smith & Nephew, Inc., Memphis, USA) supplemented by iliac crest grafting with the mixed artificial bone (Fig. 1). Postoperatively, the patient was hemodynamically stable.

**Figure 2:**
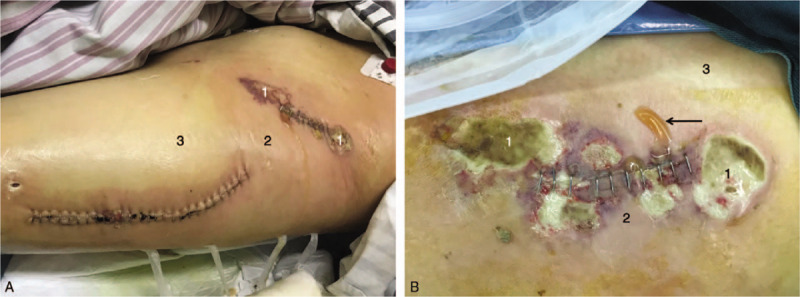
Skin manifestation and zones classification (Zone1: visible necrosis, Zone2: skin manifestations of early NF, Zone3: uninfected). (A) Red and yellow-colored lesions in both the incisions (B) Close-up anterior superior iliac spine with purulent discharge (arrow).

On day 10, the patient developed fever and weakness with extreme pain in both incisions. The lab test showed increased white blood cell (WBC) and C-reactive protein (CRP). At day 14, peri-incisional lesions have evolved and resembled cellulitis, which later became partly necrotic with discharges. The skin manifestations and zones classifications^[4]^ can be observed in Fig. 2. Based on the clinical presentations and lab test results, necrotizing soft tissue infection was suspected as a potential complication. Empiric broad-spectrum antibiotic treatment was initiated and the 6 serum parameters were collected regularly. The LRINEC scores^[3]^ were calculated and input into an Excel database (Microsoft Office Excel, 2013). Graphs with threshold scores were plotted (Fig. 3 ).

**Figure 3:**
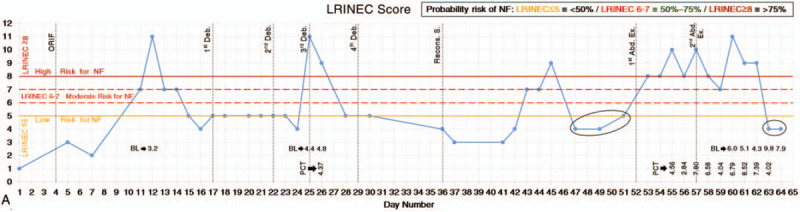
Daily variations in LRINEC score and the 6 serum parameters. (A) LRINEC score (some values of blood lactate and procalcitonin were included). (B) C-reactive protein. (C) White blood cell count. (D) Blood glucose level. (E) Hemoglobin level. (F) Sodium level. (G) Creatinine level. LRINEC = laboratory risk indicator for necrotizing fasciitis score.

**Figure 3 (Continued):**
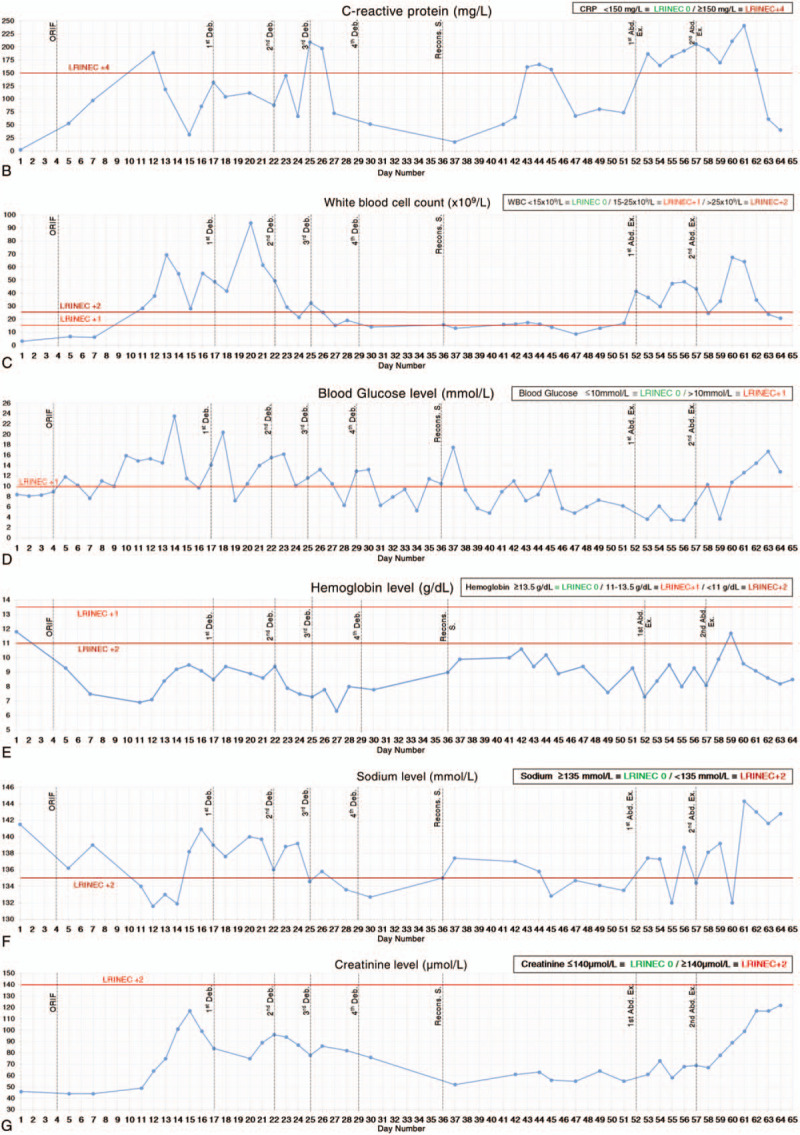
Daily variations in LRINEC score and the 6 serum parameters. (A) LRINEC score (some values of blood lactate and procalcitonin were included). (B) C-reactive protein. (C) White blood cell count. (D) Blood glucose level. (E) Hemoglobin level. (F) Sodium level. (G) Creatinine level. LRINEC = laboratory risk indicator for necrotizing fasciitis score.

**Figure 4:**
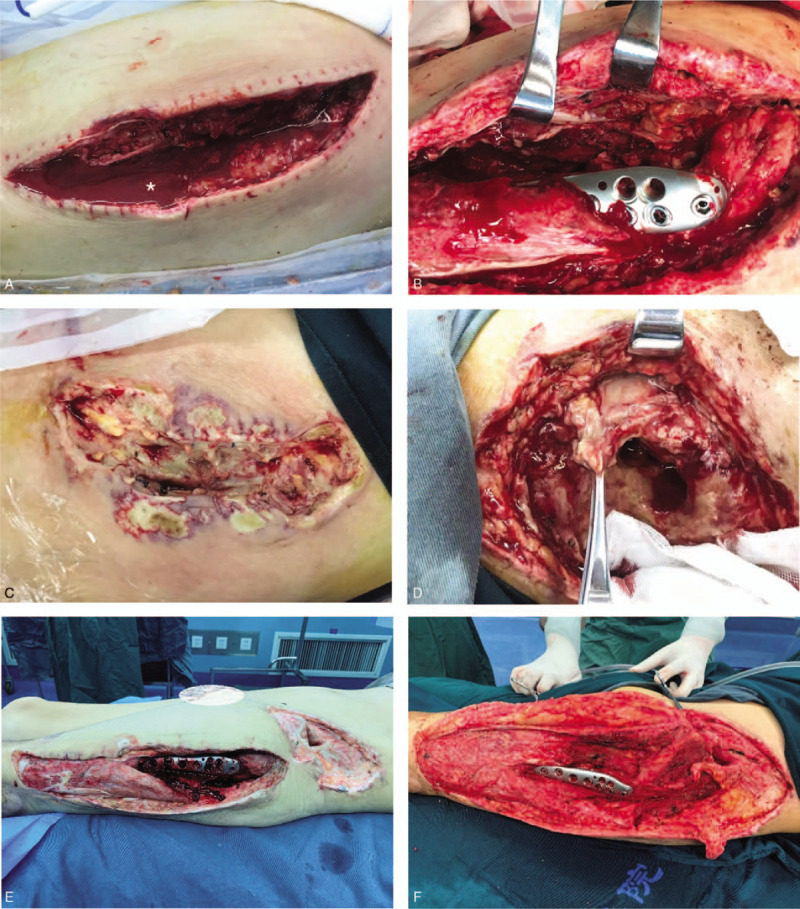
Surgical débridement. (A, B) Lateral hip wound, the first débridement revealed foul smelling grayish “dishwater pus” (^∗^). (C, D) Necrotic tissues in anterior iliac crest. E and F. The pre- and post-fourth extensive débridement, the separated wounds were connected and became extensive but cleaner.

On day 17, the diagnosis of NF was confirmed. Emergency débridement surgery with NPWT revealed grayish exudation, classic foul-smelling “dishwater pus”, a large amount of subcutaneous fat and deep fascial necrosis (Fig. 4). On day 22, 25 and 29, further radical débridements were performed. On day 22 till 42, the patient was admitted to the intensive care unit (ICU). During this period, lab test results showed that WBC, CRP, sodium, hemoglobin have declined and the LRINEC score remained ≤5 with a 2 days surge (the highest was 11). After the 4th débridement, the wound became more extensive, but it was cleaner than before. Therefore, no further débridements were necessary.

**Figure 5:**
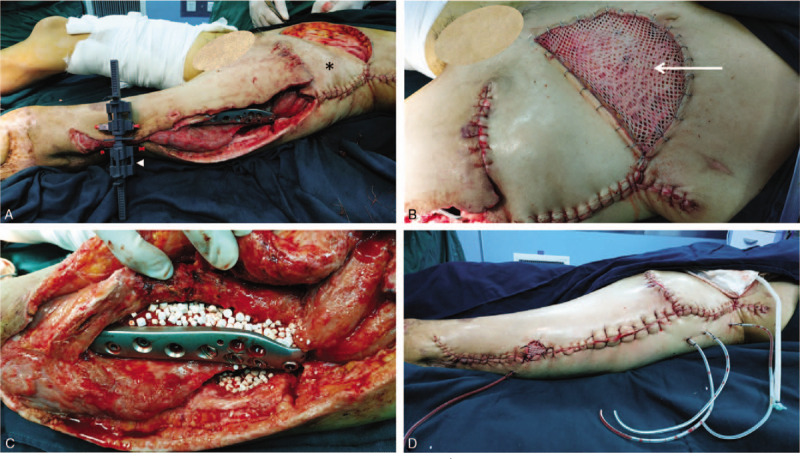
Reconstructive surgery. (A) Iliac crest covered with the rotated abdominal flap (^∗^), the skin-stretching system (arrowhead) aided in the complex wound closure. (B) Abdominal flap site was covered with the skin grafting (arrow). (C) Antibiotics-impregnated cement beads were placed around the fixed fracture with orthopedic implant. (D) The wound was successfully closed and sealed with 4 high-vacuum drainage.

On day 36, the granulation growth indicated that reconstructive surgery can be performed. The exposed iliac crest was covered by lower abdominal flap rotation. Antibiotic-impregnated cement beads (OSTEOSET Resorbable Mini-Bead Kit by Wright Medical Technology, Inc.) were placed around the exposed implant and was covered by the proximal end of tensor fascia lata rotation. Afterward, the complex wound closure was aided by using a skin-stretching system (EASApprox by BIOWIM Ltd., China), and was sealed with percutaneous high-vacuum drainage systems and NPWT (Fig. 5). For the next week, the patient was stable with no fever and less pain. WBC and CRP have leveled down to ∼15×10^9^/L and <100 mg/L, respectively. The sodium and hemoglobin levels improved, contributing to the decrease in LRINEC score to ≤4. The sutured wounds, flap and skin graft site were dry. There was no swelling or tenderness and the skin graft was fixed in uniform expansion. There was a significant overall improvement in the patient‘s condition with no signs of NF.

**Figure 6:**
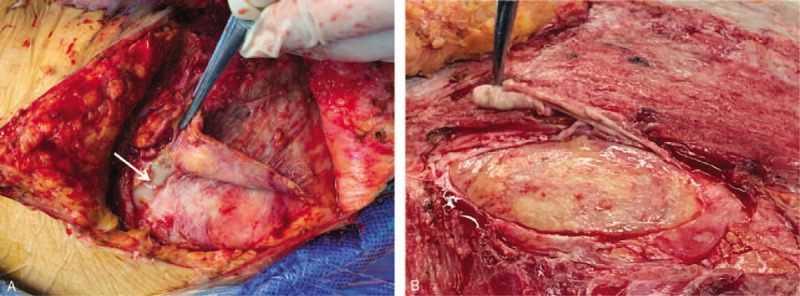
Abdominal explorations. (A) Beneath the rectus abdominis and external oblique abdominis, milky white turbid pus (arrow) was found together with deep effusion and necrosis of fascia. (B) Necrotic muscles, peritoneum and adhering greater omentum were all excised.

**Figure 7:**
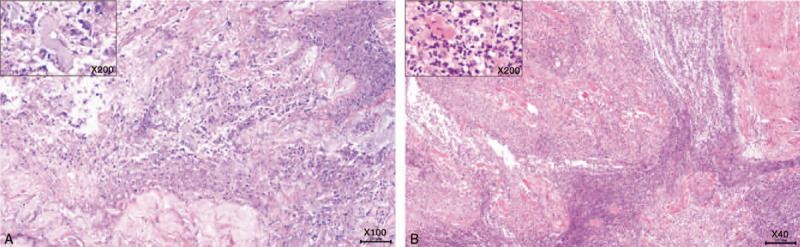
Histopathology of surgical specimens. (A) (Deep fascia of the hip) Suppurative necrotic tissue with acute and chronic inflammatory infiltration (mostly polymorphonuclear neutrophils) (X100). (B) (Rectus abdominis muscle, Peritoneum, and Greater omentum) Large amount of necrotic tissue, consistent with purulent inflammatory changes (X40).

On day 43, the patient developed recurrent high fever with a sudden increase in CRP, and LRINEC score (≥7) for 3 days, but declined to ‘low risk’ levels (LRINEC score: 4) and remained low for the next few days. WBC count remained fairly constant and low. She had an inexplicable painful subcutaneous mass in the right lower abdomen with intact skin. An abdominal computed tomography (CT) scan was nonspecific but an ultrasound showed incomplete intestinal obstruction and inflammatory lesions, which were highly suspected as recurrent NF. On day 52, emergency abdominal exploration revealed a large amount of milky-white turbid pus, effusion, deep fascia necrosis under the external oblique and rectus abdominis (Fig. 6A). Myomectomy and débridement with NPWT were performed. On day 57, another abdominal exploration was performed. The necrotic muscles, peritoneum and adhering greater omentum were all excised (Fig. 6B). Histopathological (Fig. 7) and microbiological investigations were conducted in all débridements. The results of the wound culture from the last surgery only revealed some fungal hyphae, but the fungal type was unidentified. Even though the fungal type could not be detected, the IV antifungal treatment was still administered. However, treatment did not improve the patient's condition, and the lab test results revealed a continuous increase in CRP, WBC, creatinine, LRINEC score, D-dimer, procalcitonin, lactate. The patient's instability urged her to be re-admitted to the ICU.

On day 65, her condition suddenly worsened with the presence of hypothermia, hypotension and low oxygen saturation. The conditions improved through medical interventions but remained unstable with additional signs leading to acute renal failure {↑ blood urea nitrogen (24.49mmol/L), ↑creatinine (>120 μmol/L)}. Then, atrial fibrillation appeared afterward and based on the lab results, heart failure with septic shock was suspected {↑ B-type natriuretic peptide (10.18pg/mL), ↑lactate (9.4mmol/L), ↑INR (>1.8), ↑D-dimer, ↑procalcitonin}. The patient was resuscitated and was on mechanical ventilation. Unfortunately, her condition deteriorated rapidly, and she died due to septic shock and multiple organ failure.

## 3 Discussion

Idiopathic NF remains one of the most difficult complications to be treated by physicians. The radiography (X-ray or CT scan) and microbiology findings were nonspecific in this case. However, when NF was still confined to the lower extremity, the clinical manifestations, laboratory investigations (including the LRINEC score with the 6 serum parameters), débridements and histopathological findings were crucial in the diagnosis. However, several days after the reconstructive surgery, the patient unexpectedly complained of unbearable abdominal pain and a high fever with a sudden rise in the LRINEC score, but abruptly returned to ‘lower risk’ levels after being given all the related managements. Furthermore, there was no leukocytosis, however, the hemoglobin level kept fluctuating in a decreasing manner. The clinical manifestations and laboratory findings became ambiguous and CT scans still revealed nothing. One possible explanation is that the patient‘s management of hemodynamic imbalances may affect the accuracy of the laboratory findings and LRINEC scores.^[3]^ Alternatively, ultrasound incidentally revealed the emergence of NF in the abdomen which explained the severe abdominal pain. Previous literature does suggest that the ultrasound may play a significant role in diagnosing NF, but further investigations are still needed.^[8]^ Subsequently, high levels of blood lactate, blood urea nitrogen and creatinine are all the indicators of metabolic lactic acidosis and acute renal failure which can be the important determinants for mortality in idiopathic NF as also reported by other studies.^[9]^

Since NF spreads through the superficial tissue beneath the skin, it is difficult to determine the boundary of the infected fascia. In a research study, Wong CH et al^[4]^ classified the infected skin into 3 zones: zone 1 is for the apparent necrosis, zone 2 is for the early skin manifestations of NF, and zone 3 is for the clinically uninfected skin. The classification by zones is helpful to assist the radical débridement, which aims to eradicate all of the infected tissue with the least amount of surgeries. As a result, it slows down the progression of NF while still preserving healthy tissue. Although being lifesaving, the extent of necrotic tissues that is required to be radically debrided may also bring significant challenges in wound care, functional preservation, and reconstruction which is cosmetically acceptable.^[10]^ It is noteworthy that surgery may not always be beneficial due to the inevitable loss of extensive tissue and blood. In some particular cases, the toxins can even infiltrate the systemic circulation leading to serious hemodynamic instability, multiple organ dysfunction, and septic shock. Therefore, careful management is critical for the extensive open dermofasciectomy wounds after radical débridement.^[11,12]^ According to the literature, NPWT has been proven to be beneficial in cases of NF. It helps to isolate the wound, increase tissue perfusion, decrease edema, prevent further microorganism infiltration by diminishing toxin absorbance, decrease wound dressings, control the pain, reduce the use of narcotic substances and facilitate the subsequent reconstructive surgery.^[13]^

The most distinctive feature of this multidisciplinary case is the emergence of NF in the abdomen. A latent infection might have originated from the initial lower leg wound, then migrated through hematogenous spread to the hip area where the NF developed which eventually spread to the abdomen. It is also noteworthy that the risk of developing surgical site infection still exists despite the extra intraoperative precautions. The literature reports that most of the secondary NF cases are polymicrobial, whereas idiopathic NF cases are more likely monomicrobial.^[14,15]^ Since the patient showed signs of systemic infection, a high dose of broad-spectrum antibiotics was maintained and was later supplemented with antifungal treatments. The results of the wound and blood cultures were continuously negative, which could be due to the long-term antibiotic use or an aseptic type of NF which is idiopathic in nature. Besides, the exact causative agent and the pathogenesis of NF remained unknown in this case. In the literature, idiopathic NF has been reported to possibly occur in the absence of a known causative agent or no obvious explanation for infection which may be associated with impaired immunity.^[16,17]^ Nevertheless, the combination of high dose broad-spectrum antibiotics and the débridements with NPWT contributed significantly to controlling the spread of NF. Besides, the antibiotic-impregnated cement beads placed in the areas less-accessible by IV antibiotics, especially along the fracture line (Fig. 5C) provide regional asepsis and further prevent the spread of NF by confining it in the lower extremity. Fortunately, the clinical manifestations, serum parameters, LRINEC score, and wound conditions did improve significantly. But despite all these measures, further spreading still could not be completely ruled out. This might be due to the disruption of the fascial planes owing to extensive open dermofasciectomy, which might also have allowed for more aggressive infection without elevation of inflammatory markers.^[18]^

One prospective study by Rajput et al^[19]^ discussed the roles of the advanced age, DM, abdomen involvement and late presentations in increasing mortality risk. Age over 55 years old and DM are more significant in idiopathic NF than in secondary NF.^[15]^ The patient's long history of DM with uncontrollable blood glucose levels also contributed to the declining of the body‘s immune system, thus weakened the patient's strength for recovery. The exact pathogenesis of DM in NF remains unclear. There are a few works of literature that propose several mechanisms, such as peripheral sensory polyneuropathy that may increase susceptibility to minor trauma, diabetic vascular disease that may cause tissue hypoxia and the underlying immunodeficiency.^[20]^ Even though there is considerable evidence that strongly relates DM to the pathogenesis of NF, its role as a predisposing factor for increased mortality rate is still controversial.^[21]^ Moreover, once the abdomen was involved, the NF would become uncontrollable and challenging to treat. Despite the fact that the guideline-based treatments are implemented in such a case, the possibility of the patient's improvement and survival is extremely low.

Moreover, it was further challenging for the orthopedic surgeons to decide whether it was necessary to remove the hardware of the internal fixation in the presence of NF. The related literature is scarce and there is no evidence-based guideline for such a case with an unstable fracture. Despite vigorous débridements, the risk of possible microorganisms that might be present between the hardware and the fractured bone cannot also be ruled out. In a study by Worlock et al^[22]^, the diaphyseal tibial fractures were surgically fixed either with an unstable endomedular pin or with a stable compression plate. They introduced inoculated Staphylococcus aureus in the fracture zones to investigate the infectious state from both surgical methods. It was shown that the unstable group was 2 times more infectious than the stable compression plate group (71% vs 35%). Berkes et al^[23]^ concluded that deep infection after ORIF can be treated with débridements, antibiotic treatments, and the retention of hardware until fracture union is achieved. The stability provided by the implants has been proven to help not only in reducing the incidence of infection after internal fixation but also the clearance of established infections.^[24,25]^ Therefore, it is highly recommended to maintain the fixation until osseous union has been achieved.

## 4 Conclusion

The clinical course of idiopathic NF in polytrauma post-ORIF patients with DM is unpredictable and is associated with a high risk of mortality. Patients should be monitored closely in the ICU. A multidisciplinary approach is encouraged for urgent decision making and close monitoring. Possible fascial spread to the abdomen without specific clinical manifestations need to be anticipated. Early intervention, aggressive radical débridement with NPWT, broad-spectrum antibiotics and hemodynamic support are the mainline treatments of NF. The possible underlying systemic diseases and the source of infection should be addressed and treated appropriately. We recommend the placement of antibiotic cement beads in the case of NF-complicated post-ORIF of the lower limb without the removal of hardware. Daily measurement of LRINEC scores with graphs helps to obtain better visualization and to detect early trends of unusual courses. The NF-complicated ORIF cases which re-emerge in the abdomen still require further investigations.

## Acknowledgments

We would like to thank Ghamor-Amegavi Edem Prince for his assistance; and Jinwu Bai for the ethical committee procedures. The persons who were acknowledged have given permission to be named.

## Author contributions

**Conceptualization:** Muhammad Umar Joomun, Deting Xue

**Investigation:** Muhammad Umar Joomun, Zhiya Li

**Methodology:** Deting Xue, Huawei Shao

**Supervision:** Zhijun Pan

**Validation:** Zhijun Pan

**Writing – original draft:** Muhammad Umar Joomun, Zhiya Li

**Writing – review & editing:** Muhammad Umar Joomun, Zhiya Li, Deting Xue, Huawei Shao, Zhijun Pan
